# Feasibility of Using Bioelectrical Impedance Analysis for Assessing Youth Weight and Health Status: Preliminary Findings

**DOI:** 10.3390/ijerph181910094

**Published:** 2021-09-26

**Authors:** Cheryl A. Howe, Riley J. Corrigan, Maya Djalali, Chris McManaway, Alexandra Grbcich, Grace Sam Aidoo

**Affiliations:** 1 School of Applied Health Sciences and Wellness, Ohio University, Athens, OH 45701, USA; cm065418@ohio.edu (C.M.); ag792712@ohio.edu (A.G.); ga985018@ohio.edu (G.S.A.); 2 Honors Tutorial College, Ohio University, Athens, OH 45701, USA; rc401218@ohio.edu (R.J.C.); md169519@ohio.edu (M.D.)

**Keywords:** children, adolescents, body composition, cellular integrity, bio-electrical impedance analysis

## Abstract

*Background.* This study assessed the accuracy of bioimpedance analysis (BIA) for measuring body composition and resting metabolic rate (RMR) in fasted and non-fasted state and the prospect of using phase angle (PA) to indicate cellular health in youth. *Methods.* BIA body composition, RMR, and hydration measures were compared to dual-energy *x*-ray absorptiometry (DXA), MedGem metabolic analyzer, and urine specific gravity, respectively, at baseline in a fasted state using one-way ANOVAs. Repeated BIAs at 0, 30, 60, 90, and 120 min post-prandial were compared to baseline using repeated-measures ANOVA. Correlations were used to assess the relationship among PA and health (blood lipids and glucose, resting BP) and fitness (grip strength and a 3 min step test) measures. *Results.* BIA scans (N = 58; 11.4 ± 2.9 y) measured lower body fat % (BF%) in healthy weight youth (BMI < 85th percentile; 16.4 ± 1.1 vs. 25.1 ± 1.0%) and lower visceral adipose tissue (VAT) in males (44.5 ± 2.9 vs. 34.1 ± 6.0 cm^2^) than DXA and higher RMR in all youth (1244 ± 41 vs. 1104 ± 39 kcals/day), healthy weight (1231 ± 48 vs. 1049 ± 44 kcals/day), and teens (1541 ± 62 vs. 1234 ± 72 kcals/day) than MedGem. Compared to baseline, immediate post-prandial values were significantly higher for BF% (21.4 ± 1.4 vs. 22.0 ± 1.4%) and VAT (45.4 ± 6.1 vs. 46.2 ± 6.2 cm^2^). PA was significantly correlated with BF% (r = −0.33; *p* = 0.01), fat-free mass (r = 0.59; *p* < 0.001), grip strength (r = 0.56; *p* < 0.001). *Conclusions.* While more data are needed to confirm these preliminary findings, the results suggest caution is necessary in using BIA to assess aspects of youth health and weight status, especially in males, healthy weight, and teens. However, these preliminary findings do indicate that phase angle maybe be a valuable, non-invasive tool for identifying youth who are heading towards obesity and/or obesity-related health consequences.

## 1. Introduction

Attention on youth health and wellness in the United States is due mainly to the steady incline of obesity rates. In fact, 18.5% of U.S. children are considered obese, a statistic that has more than tripled over the last four decades [1,2]. Childhood obesity is concerning as it routinely persists into adulthood, which can lead to earlier onset of chronic diseases typically seen only in adults, such as cancer, cardiovascular disease, and type 2 diabetes [1,3]. In order to understand the weight of these concerning facts, one must understand the definition of obesity that is accepted in the United States.

According to the National Health and Nutrition Examination Survey (NHANES), a cross-sectional survey that represents the population widely accepted as a credible indicator of health trends in the United States, obesity in children aged 2 to 19 years is defined as having a body mass index (BMI) at or above the 95th percentile according to the CDC’s age- and sex-specific growth charts [4]. While BMI, a calculation of weight relative to height, is considered an index for determining weight status, there is reason to question whether there may be better indicators, mainly because BMI does not account for the individual components of weight, such as fat mass (FM) or fat-free mass (FFM). A recent study of 8-year-old children demonstrated the variability of body compositions at the same BMI value [5]. Specifically, children with the same BMI had very different adiposity levels, and similarly, those with similar body fat percentages (BF%) had very different BMI values. These findings question the validity of BMI and support the need for alternative methods for determining body composition, not BMI in this population.

Body composition can be measured in many ways, with each method possessing pros and cons and different sources of error. While dual-energy *x*-ray absorptiometry (DXA) is becoming the gold standard and most widely used as a criterion measure in validating other methods [6], the cost and accessibility of this device as well as the radiation exposure are barriers to using DXA in locations such as schools and pediatrician offices for the purpose of more accurately measuring and tracking youth obesity rates [7].

Bioimpedance analysis (BIA) offers a more accessible and simplistic method of measuring body composition and has been shown to provide accurate measures among adults and children for clinical and epidemiological purposes [8]. This method is premised on the distribution and quantity of tissues with varying electrical properties. For example, FFM is primarily composed of water and electrolytes and acts as a good conductor (low impedance) of electrical current, while FM is anhydrous and a poor conductor (high impedance); the larger the FM the higher resistance it poses to an electric current [9]. Thus, BIA measures the voltage drop as electric currents move through segments of the body and the capacitance created by cell membranes [10,11]. Estimations of total body water are used to calculate FFM, while FM is calculated by subtracting FFM from total body weight.

One of the issues that compromises the accuracy of older-generation BIA models which use a single frequency is the impact of hydration status and food intake on these results since dehydration can slow the currents movement through the body. The InBody 770 BIA (InBody USA, Cerritos, CA, USA) has the capacity to monitor body fluids as well as nutritional status of individuals, where nutritional status accounts for an individual’s health condition as influenced by the intake and use of nutrients, including water [12]. A study by Saunders et al. (1998) found that fluctuations in hydration status altered the BIA-derived BF% in athletes (N = 15; 19–56 y) by as much as 2–3% [13]. States of dehydration reduce resistance of the electrical current resulting in the presumption of lesser FFM. This finding was supported in a study that induced dehydration with diuretics in healthy weight men (N = 18; 23–47 y) to find BIA-derived FFM decreased by 2.63 kg [14]. Similarly, a more recent study (N = 100; 24.2 ± 6.7 y) also found that consuming 500 mL of water prior to a BIA assessment resulted in a significant decrease in BF% of 0.16% (*p* = 0.02) [15].

Newer-generation BIA models, such as the InBody 770, use the capacitive properties of the body to estimate total body water, thus resolving one of the main criticisms of previous models—not accounting for hydration status. These capacitive properties of the cell indicate the overall health of the cell. Specifically, the greater the ability of the cell membrane to store electric charge, the greater the overall health of the cell [16,17]. The InBody 770 also incorporates six different frequencies (1, 5, 50, 250, 500, and 1000 kHz) and a tetrapolar eight-point electrode system to assess the entire body, including visceral adipose tissue (VAT). Previous research has validated different BIA models against DXA in children. A recent review found that BIA-derived whole-body BF% in children was comparable to DXA values (r values ranging from 0.75 to 0.92) when using multiple frequencies and multiple electrodes on both side of the body, whereas the correlations between DXA and segmented FM and FFM values were more varied by BIA model (r= 0.14 to 0.88) [18]. In both boys and girls, 62.5–66.7% of the studies (N = 30) underestimated measures of FM compared to a criterion reference.

The InBody 770 uses the phasal shift, or phase angle (PA) of the current moving through the body to determine cellular health. PA reflects the resistance and reactance of the body’s cells in response to the external current, with the healthier cellular membrane causing a greater phasal shift, thus a greater PA. PA is used to assess disease progression or recovery in those with chronic disease in youth [19,20]. For example, children (N = 67, 3–20 y) undergoing treatment for bone marrow disfunction exhibited lower PA values compared to healthy controls and these values decreased further in response to the aggressive treatment [19]. In this sense, PA is being used as a prognostic tool. Further, this study found that without concomitant changes in cell mass related to the treatment, changes in PA indicate a sensitivity to cell integrity, regardless of changes in body mass. Similarly, PA has been used to determine recovery progress in children (N = 122, 0–16 y) undergoing cardiac surgery [20]. In this study, a PA of ≤2.7° at 2 days post-surgery was indicative of a longer length of stay in the hospital, an indicator of survival from surgery. These studies demonstrate that PA is a valuable, non-invasive tool for assessing disease progression and recovery. However, there is little research to determine if PA could be used to prevent disease, especially in the obese youth. If PA is sensitive to damage to cell membrane integrity and cell death, which leads to inflammation and inflammatory diseases [21], clinicians could detect health issues and intervene earlier to prevent obesity and obesity-related chronic diseases.

The purposes of this preliminary study were trifold. First, this study assessed the accuracy of body composition and basal metabolic rate (BMR) measures from the InBody 770 BIA against criterion measures. While the ease of using this device is valuable, the accuracy in youth is not yet confirmed. If found to be accurate against the criterion measures, this device could be incorporated into schools and clinics as a more accurate assessment of weight status as well as provide a starting point for determining appropriate energy intake requirements for treating or preventing obesity. Second, this study assessed the impact of fasting status on InBody 770 measures of FM, FFM, BF%, VAT, BMR, and PA. Placing this device in schools and clinics would provide valuable information to parents or practitioners, but fasting status is difficult to control in these situations. The research in this area is inconsistent in children. Understanding the impact of fasting status could provide guidance on the timing of BIA scans. Lastly, this study correlated measures of health (cardiovascular disease risk factors) and fitness (cardiorespiratory fitness and muscular strength) with PA measures from the InBody 770. The InBody 770 is not limited to measures of body composition and RMR but can also provide a non-invasive measure of cellular health that has the potential for early detection and prevention of chronic disease in youth. Until now PA has been used in a clinical sense to monitor disease prognosis, progress, and recovery. The hope is to use this tool to identify children who require intervention for the prevention of obesity and obesity-related diseases. However, for this to be possible, reference values for apparently healthy youth of varying sex, age, and weight status need to be established.

## 2. Materials and Methods

**Recruitment.** Youth (7–17 y) were recruited from Athens and surrounding counties in Ohio. Youth were considered eligible if they were physically capable of completing a moderate exercise bout and were free from musculoskeletal, neurological, and physiological conditions that could be made worse with exercise. Both the parent or guardian and the participant provided informed consent and assent, respectively. This study was approved by Ohio University’s Institutional Review Board.

Eligible youth were asked to prepare for a single testing session by (1) avoiding all strenuous exercise/activity for at least 12 h; (2) abstaining from eating or drinking anything, except unlimited water for 3 h; and (3) wearing gym attire without metal zippers or plastic buckles as these could interfere with the measurements.

**Anthropometrics.** Height (cm) was measured to the nearest 0.1 cm using a stadiometer (Seca 213, Chino, CA, USA) both in a standing and seated position. Combined with age and sex, the standing and seated height were used to estimate maturation status as years (±) from peak height velocity using the Mirwald equation [22]. Weight (kg) was measured to the nearest 0.1 kg using the InBody 770 scale (InBody USA, Cerritos, CA, USA). Prior to stepping on the scale, participants were asked to stand for at least 5 min and to clean the palmar and plantar aspects of their hands and feet, respectively, with a wet towelette. After measuring weight, participants followed the verbal instructions given by the device to grasp both handles with their elbows fully extended and at an angle away from the body, to not talk and to not move until the measurement was complete (approximately 1 min). The InBody 770 provided overall and compartmentalized values for FM, FFM, BF%, VAT, BMR, intra and extracellular water analysis for estimating hydration status, and PA as a measure of cellular health. PA is defined as angular displacement between the current and voltage waveforms as a result of capacitor interference, measured in degrees or radians. PA is related to cell permeability and soft tissue hydration and is calculated as arctangent (reactance/resistance) × 180°/π. The BIA was conducted in a fasted state and immediately post and at 30, 60, 90, and 120 min following a meal.

**Hydration Status.** Urine samples were used to determine urine specific gravity (USG) using reagent test strips (10SG Urine Reagent strips; range: 1.005 to 1.035) and a urine analyzer (McKesson Consult™ 121, Irving, TX, USA). Measures of hydration status from reagent strips have been shown to be strongly correlated with refractometry measures of USG in young male boxers (r = 0.85; *p* < 0.05) [23] and youth soccer players (r = 0.80, *p* < 0.01) [24]. Hydration was also calculated from the BIA scan as a percentage that total body water weight (%TBW) contributes to total body mass.

**Resting Metabolic Rate.** After resting for 10 min in a recliner in a temperature-controlled room with no disturbances, participants were asked to breathe through a portable metabolic analyzer (MedGem, MicroLife^®^ Medical Home Solutions, Golden CO) for a maximum of 10.5 min to measure resting metabolic rate (RMR; kcals/day) following a 3-h fast. Since the InBody 770 provides estimations of BMR, which is approximately 10% lower than RMR, BIA values were adjusted accordingly (RMR = BMR × 1.10) for direct comparisons. The MedGem metabolic analyzer is accurate and reliable for measuring RMR in this population with a ±1.15% mean difference in RMR measures between MedGem and Douglas bag methods [25], and intraclass reliability ranging from 0.97 to 0.98 [26].

**Blood Analysis.** While in a fasted state, blood lipids and glucose levels were obtained from a fingerstick sample. A 40 µL capillary tube of blood was analyzed using a Cholestatic LDX^®^ System (Alere, Charlottesville, VA). A lipid panel, including total cholesterol (TC; 100–500 mg/dL), high-density lipoproteins (HDL; 15–100 mg/dL), triglycerides (TRG; 40–650 mg/dL), an estimation of low-density lipoproteins (LDL), as well as glucose (mg/dL) were measured with single-use cassettes. The Cholestech was calibrated as per manufacturers recommendations prior to use. The Cholestech has been found valid and reliable for these measures. Specifically, in comparing 119 patients’ samples with laboratory analysis, the correlation coefficients were r = 0.97 for TC (CV ≈ 5%), and r = 0.95 for HDL (CV = 5–10%) [27], and when compared to a venous blood sample from 250 healthy family members of cardiovascular patients, the correlations coefficients were r = 0.91, 0.88, 0.70, and 0.93 for TC, LDL, HDL, and TRG, respectively (*p* < 0.01) [28].

**Body Composition.** A whole-body dual-energy *x*-ray absorptiometry (DXA; Hologic^®^, Marlborough, MA, USA) scan was performed on all participants by a licensed and experienced general *x*-ray machine operator to determine FM and FFM and BF%. The participants removed their shoes and socks and laid supine on the DXA scanner for 6 min. The DXA software was used to process each scan by compartmentalizing the body into right and left arm and leg and trunk.

**Standardized School Lunch.** After the DXA scan, participants were offered a standard school lunch, which consisted of a main entrée, fruit, vegetable, dip, and a drink (500–600 kcals).

**Resting Hemodynamics.** During one of the free periods between post-prandial BIA scans, the participant again rested for at least 10 min in a seated position. Resting blood pressure and heart rate were measured using the OMRON digital blood pressure monitor (Omron Electronics, LLC, Estates, IL, USA) with the appropriate cuff size. The procedure was repeated to ensure at least 2 measures with less than 5 mmHg difference.

**Muscular Strength.** Grip strength (kg) was measured using a handgrip dynamometer (Jamar Hydraulic, JLW Instruments, Chicago, IL, USA) as an indicator of muscular strength. Participant were asked to stand tall with their elbow at a 90° angle looking straight ahead with their feet hip width apart to distribute their weight evenly. With the dynamometer adjusted so that their middle metacarpal formed a 90° angle, participants were asked to perform a maximal squeeze for at least 3–5 s three times in each hand.

**Cardiovascular Fitness.** During the final break between BIA scans, cardiovascular fitness was measured using the Kasch Pulse Recovery (KPR) test. Participants wore a POLAR RS400 heart rate monitor watch and strap to record heart rate at the end of each minute during the test and through at least 3 min of recovery. The test requires the participant to step up and down on a 12-inch platform for 3 min at a pace of 24 steps per minute set by a metronome. Immediately post-exercise, participants were asked to sit down on the platform and heart rate was recorded after each minute of the recovery period. The 1 min recovery heart rate was used to estimate maximal oxygen consumption (Est.VO_2_max; mL/kg/min) as a measure of cardiovascular fitness. The KPR has been validated for use in this population for estimating cardiovascular fitness [29,30].

**Statistical Analysis.** Means (±SD) were calculated for all participant characteristics and measurements. FM and FFM were also expressed relative to height (kg/m^2^); FM index (FMI) and FFM index (FFMI) for both BIA and DXA measures. Only whole-body measures are reported in this manuscript, the compartmentalized values were not reported or analyzed. One-way ANOVA was used to compare all BIA measures to the criterion measures overall and by sex, age group (child, 7–12 y; teen, 13–17 y), and weight status [healthy weight (HW), BMI < 85th percentile; overweight (OW), BMI ≥ 85th and <95th percentiles; obese (OB), BMI ≥ 95th percentile]. Repeated-measures ANOVA with Bonferroni adjustment was used to compare the BIA measures between fasted and non-fasted status from baseline to 120 min post-prandial. Correlation analyses were used to assess the relationship among total body water relative to lean body mass (TBW/LBM), %TBW and USG, and the relationship among PA and resting blood pressure, blood lipids and glucose, grip strength, and estimated VO_2_max. All statistics were performed using SAS Enterprise Guide (version 7.1) with significance set at *p* < 0.05.

## 3. Results

### 3.1. General Characteristics

To date, 58 children (30 girls; 37 HW) participated in the Ohio University BIA Validation Study. The general characteristics for the sample overall and by group (sex, weight status, and age group) are presented in Table 1. As expected, teens were heavier and taller than the younger children and there was a significant difference in BMI (kg/m^2^ and percentile) across weight status. Teens also had 19.3% higher RMR than younger children (*p* < 0.05). Health and fitness characteristics of the sample are also presented in Table 1 overall and by group. Sex and weight status had no significant effect on health and fitness measures. However, teens had approx. 10.9% lower TC, 17.8% lower HDL and 3.5% lower Est.VO_2_max, 6.1% higher systolic BP, and 49.3% greater grip strength compared to the younger children (*p* < 0.05). DXA scans revealed that BF% was significantly higher in girls (+18.2%, *p* = 0.03) and children (+24.9%, *p* = 0.005) compared to their counterparts. The DXA-derived FM, FMI, VAT, and BF% were significantly different across weight classifications as expected, but FFMI was also 13.8% higher in OB compared to HW youth and 16.1% higher in teens compared to children. Baseline BIA scans revealed that PA was 5.5% (*p* = 0.04) higher in boys than girls while BF% was 29.1% (*p* = 0.04) higher in girls. Teens had 32.7% higher BIA-derived BMR, 12.6% higher PA, 46.8% higher FFM and 14.7% higher FFMI than younger children (*p* values < 0.0001). All fat-related parameters (FM, BF%, VAT, and FMI) were significantly different across weight classifications, as expected, but FFMI also increased by 8.0% from HW to OB youths (*p* < 0.0001).

### 3.2. Baseline Comparisons between BIA and Criterion Measures

Comparison between BIA values and the respective criterion measures are presented in Table 2. Overall, there were no significant differences between DXA and BIA measures of body composition when the sample was analyzed as a whole and by sex and age group. The results were inconsistent when analyzed by weight status, with BIA measuring BF% 42.0% (*p* < 0.001) lower in HW youth compared to DXA. After converting BMR to RMR, BIA-derived RMR was significantly higher (*p* < 0.001) than MedGem values, overestimating RMR by 12.4% (+148 kcals/day) compared to MedGem values. Specifically, these BIA-derived RMR values were higher in girls (+15.1%, *p* < 0.001), boys +10.0%, *p* = 0.002), HW (+16.9%, *p* < 0.001), children (+7.6%, *p* < 0.001), and teens (+10.3%, *p* < 0.001) compared to MedGem.

Since there was no direct comparison between USG and measures of hydration (body water analyses) from the BIA, correlation coefficients were used to determine consistency between methods. USG was not well correlated with any measure of body water content from the BIA, including %TBW (r = −0.25, *p* = 0.07) and TBW/LBM (r = 0.25, *p* = 0.07) overall or by age group. However, there was a moderate correlation between USG and %TBW for boys (r = −0.48, *p* = 0.02) and strong correlations among USG and TBW (r = −0.86, *p* = 0.006) and TBW/LBM (r = 0.82, *p* = 0.02) for OW youth.

### 3.3. Fasting vs. Non-Fasting State

One participant declined participation in the repeated-measures portion of this study, only completing the baseline comparisons. An additional five participants did not complete the final BIA scan at 120 min due to time constraints, thus accounting for the differences in sample size across timepoints. Comparison of baseline to post-prandial BIA measures are presented in Table 3. BIA measures across timepoints were not consistent. Specifically, weight was held constant throughout the six scans, while BMR did not differ between baseline and all post-prandial measures, but at 90 (+0.3%, *adj. p* = 0.008) and 120 (+0.1%, *adj. p* = 0.011) minutes BMR was higher than immediate post-prandial (0 min). Similarly, PA did not differ between baseline and any other timepoint, but PA was significantly lower at 120 min compared to 30 (0.9%, *adj. p* = 0.008) and 60 (0.7%, *adj. p* = 0.012) minutes post-prandial. With respect to the FM analysis, BF% was significantly different at baseline compared to all timepoints (range: +2.5 to −3.9%, *adj. p* values < 0.05) and VAT measures were higher at 0 (+1.7%, *adj. p* = 0.006) and lower at 120 (−10.9%, *adj. p* = 0.014) minutes for VAT compared to baseline measures. FFM analysis revealed again that there were no differences between baseline and all other timepoints, but immediate post-prandial was lower than at 90 (0.5%, *adj. p* = 0.013) and 120 (0.1%, *adj. p* = 0.017) minutes post-prandial. While there was no difference in %TBW, defined as the amount of body weight accounted for by water weight and an indicator of hydration status across all timepoints, the other measures of body water differed significantly across many of the timepoints.

### 3.4. Relationship among PA and Health and Fitness Measures Overall

**Figure 1:**
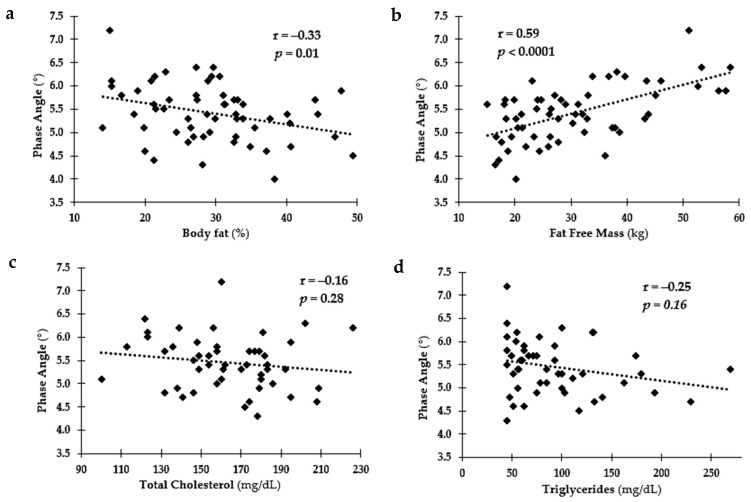
Relationship among phase angle (PA) and health status. (**a**) PA vs. body fat (%); (**b**) PA vs. fat-free Mass (kg); (**c**) PA vs. Total Cholesterol (mg/dL); (**d**) PA vs. Triglycerides (mg/dL).

**Figure 2:**
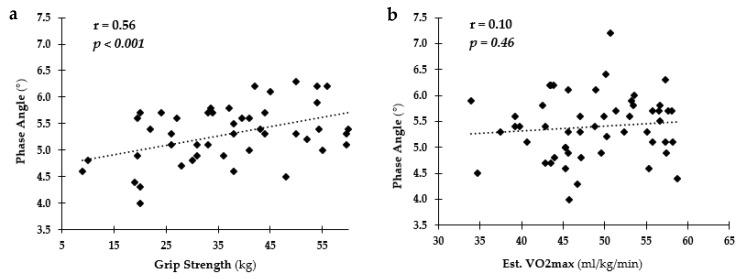
Relationship among phase angle (PA) and fitness levels. (**a**) PA vs. grip strength (kg); (**b**) PA vs. Est. VO_2_max (mL/kg/min).

Figure 1 depicts the relationships among PA and body composition measures (BF% and FFM) and blood analysis measures (TC and TRG). A negative relationship was found among BF%, TC, TRG, and PA with a positive relationship between PA and FFM. The relationships among PA and fitness levels (grip strength and Est. VO_2_max) are depicted in Figure 2. Positive relationships were observed for PA and both fitness measures.

## 4. Discussion

The purpose of this preliminary study was to assess the overall accuracy and feasibility of using BIA for measuring and tracking weight and health status in youth of varying age, sex, and weight classifications. Recruitment was stratified to include even samples of older and younger, healthy weight, overweight and obese girls, and boys. In this preliminary paper, some of the subgroups, while small have adequate power for simple comparisons, but are not large enough to assess age, sex, and weight classification interactions. This will be included in the final analyses.

**Baseline Comparison.** The new-generation BIA device is simple to use, portable and accounts for hydration status, which has been shown to be a significant source of error [8]. Overall, the findings are unequivocal in some respects, but show promise in others. For example, in comparing the BIA-derived whole-body measures of body composition to DXA, there was no difference in BF%, FMI, FFMI, and VAT when assessing the group as a whole. However, BIA was significantly different for %BF, FMI, and FFMI in HW youth and FMI in teens. All other body composition measures were not significantly different between methods. The correlation coefficient for %BF was r = 0.96 (*p* < 0.0001) between DXA and BIA and for RMR was r = 0.79 (*p* < 0.0001) between BIA and MedGem. Previous research comparing InBody 720 BIA to DXA have also found strong correlation coefficients in this population ranging from r = 0.80 to 0.99 [18,31,32], with one study (N = 178, 7.7 ± 0.4 y) also reporting very similar %BF in both boys (r = 0.94) and girls (r = 0.95) [32]. Similarly a study comparing the Tanita BF-689 BIA (pediatric lower body device) to DXA reported a strong intraclass correlation coefficients (ICC) for %BF of 0.788 (95% confidence interval: −0.167, 0.942), which did not change when conducting sex-specific ICCs [boys 0.786 (−0.182, 0.944) and girls 0.764 (−0.163, 0.939)] [33]. The mean difference found between Tanita and DXA of −6.75% was similar to the mean difference of 7.8% between InBody and DXA found in the current study.

Even though some values were significantly different, care should be taken when relying too heavily on statistical significance as some comparisons may not be physiologically or practically meaningful or vice versa. For example, BIA-derived VAT in OB youth was 29.1% higher than DXA. Although not statistically different (*adj. p* = 1.00), an overestimation of VAT could exaggerate health risks association with this type of body fat. In contrast, BIA-derived BF% was 37.1% lower than DXA, which would have resulted in a lower body fat classification for the overall sample. Such an underestimation is physiologically meaningful since it could miss identifying youth who are at risk for obesity-related complications. More research is needed with larger samples to see if these differences persist.

This is also noted when examining the differences in RMR between BIA and the MedGem metabolic analyzer. This appears to be the first study to compare RMR values between these devices in this population. While BIA was significantly higher than MedGem-derived RMR overall and for HW youth and teens, the mean difference was only +170.8 kcals/day. Practically, this overestimation could lead to a surplus intake of 1195 kcals/week. Since the childhood obesity problem is associated with small chronic energy surpluses, found to be as little as +15 kcals/day in 2–7 y children [34,35], the magnitude of this surplus could contribute rather than help alleviate the childhood obesity problem.

**Fasting vs. Non-Fasting.** The second purpose of this study was to assess the impact of fasting status on BIA measures. Ideally, since BIA is quick, simple to use and places very little burden on the child, it has the potential to replace standard weight scales in schools and pediatrician offices for measuring and tracking weight and body composition. However, measuring body composition in these places does not guarantee the child would be in a fasted state as recommended by the manufacturer (InBody USA). Adult research has demonstrated that feeding decreased impedance measured by BIA by 4.4%, leading a decrease in FM (−1.8%) and BF% (−2.5%) in young adults (N = 54; 21.8 ± 2.9 y; 27 males) [36]. In that study, non-fasting status accounted for 37 to 79% of the variability of the measures. In the current study, body weight, FM, FFM, BMR, and water analyses did not differ from baseline following a standard school lunch. However, for the first 60 min post-prandial BF% was significantly higher from baseline but this difference disappeared for the last two measures (90 and 120 min). The mean difference from baseline across sex, age, weight status varied from −3.0 to +3.3% across post-prandial timepoints. These differences are negligible and would likely result in few misclassifications of obesity status based on BF%. Similarly, changes in BIA-derived BMR from baseline across sex, age, and weight status varied from −34 to +26 kcals/day throughout the 2 h of post-prandial measures. While more research is needed to determine if weight status classifications would be affected by fasting status when using BIA to measure BF%, the information about nutritional needs (BMR measure) appears to closely match measures from the MedGem metabolic analyzer (RMR measures). This would facilitate appropriate nutritional counselling for obesity treatment and prevention.

The biggest concern about the effects of feeding prior to BIA measures was the variability in the body water analyses. While TBW and ECW/TBW (a measure of extracellular water relative to total body water) between baseline and several timepoints, the main measures provided by the InBody 770 used for determining FM and FFM (TBW/LBM and %TBW) did not differ from baseline. These findings show promise for using the InBody 770 BIA for measuring and tracking body composition and nutritional status in children.

The final purpose of this study was to assess the sensitivity of PA to differences in health and fitness levels in youth. Significant relationships were found among PA and health and fitness levels. Specifically, cardiometabolic disease markers, such as TC, TRG and BF%, were negatively associated with PA; lower TC, TRG, and BF% were related to higher PA values indicating healthier bodies and therefore, healthier cells. There was also a positive relationship found among FFM and fitness markers (grip strength and Est. VO_2_max), especially in OW and OB youth. As expected, increased strength and FFM would be related to better fitness and therefore, healthier bodies and cells. PA is dependent on the opposition to the flow of the electrical current (resistance) and the capacitive ability of the cell membrane to impede the current (reactance) [20]. Meaning, healthier cells will result in a greater phasal shift and thus a greater PA compared to less healthy cells as seen in these findings. In previous research, changes in PA have been shown to precede changes in body weight and as such may be an early marker of changes in an individual’s overall resilience or health [19,37,38]. Most research on PA in children have focus on malnutrition related to disease or illness. This is one of the first to examine if PA could detect variations in health and fitness in otherwise healthy youth and to establish healthy ranges for these measures across sex, age, and weight status. These findings indicate PA might be a valid measure of cellular health and could be used to identify children heading toward chronic disease.

As all studies have limitations, this study is limited in sample size, especially in OW and OB youth and teens. Based on these preliminary findings, recruitment for this study has continued in order to increase sample size and balance the subgroups. This will allow for more sophisticated statistical analyses, such as two- and three-way comparisons to assess group and timepoint interactions as well as multiple regression analyses. Future research will investigate changes in BIA-derived measures in youth over time in schools, pediatrician offices, and nutrition clinics. This longitudinal study would test if this simple, non-invasive device can be used for early detection of excessive growth patterns that could lead to early onset of chronic disease in youth.

## 5. Conclusions

These preliminary findings suggest some caution is necessary in using BIA to assess youth health and weight status, especially in boys, healthy weight youth and teens. However, more research is necessary in larger sample sizes to confirm these findings and to determine the practicality and feasibility of using such a device in schools and clinics for measuring and tracking body composition in youth, although the non-invasive phase angle measure does show promise as a marker for early detection and prevention of childhood obesity and obesity-related chronic diseases.

## Figures and Tables

**Table 1:** Participant characteristics by sex, weight status and age group (means ± SD).

	Sex		Weight Status			Age Group	
Sample Size	Girls	Boys	HW	OW	OB	Child	Teen
	29	29	37	10	11	39	19
Anthropometrics							
Age (years)	11.1 ± 2.6	11.7 ± 3.2	11.8 ± 3.0	11.1 ± 2.9	10.4 ± 2.7	9.7 ± 1.7 *	14.8 ± 1.5 *
Weight (kg)	46.3 ± 18.4	48.0 ± 18.2	43.1 ± 14.2 *	**48.2 ± 16.2**	59.8 ± 26.3 *	41.3 ± 17.7 *	59.1 ± 12.8 *
Height (cm)	147.3 ± 14.3	150.1 ± 19.6	150.0 ± 17.7	147.0 ± 17.1	145.7 ± 15.9	140.3 ± 12.6 *	165.9 ± 11.0 *
BMI (%ile)	65.9 ± 27.9	67.0 ± 32.2	51.4 ± 27.5 *	88.2 ± 2.1 *	97.3 ± 1.4 *	72.0 ± 28.2 *	55.1 ± 30.8 *
RMR (Kcals/day)	1042 ± 210 *	1202 ± 308 *	1068 ± 257	1209 ± 251	**1231 ± 312**	1043 ± 232 *	1267 ± 290 *
Blood Analysis							
TC (mg/dL)	165.2 ± 5.0	161.1 ± 5.9	158.6 ± 4.7	169.6 ± 9.9	174.5 ± 6.6	168.9 ± 22.7 *	151.5 ± 30.6 *
HDL (mg/dL)	52.0 ± 2.7	53.1 ± 3.6	54.7 ± 3.0	51.0 ± 4.8	46.1 ± 2.7	55.6 ± 16.0	46.5 ± 12.4
TRG (mg/dL)	97.0 ± 10.2	85.0 ± 10.6	83.9 ± 8.0	98.8 ± 19.2	114.1 ± 22.8	100.2 ± 57.0	73.9 ± 30.3
LDL (mg/dL)	94.1 ± 5.5	90.7 ± 4.7	87.3 ± 4.8	98.7 ± 6.9	105.0 ± 6.5	93.2 ± 25.2	90.3 ± 26.5
Glucose (mg/dL)	83.2 ± 1.7 *	91.0 ± 1.3 *	85.8 ± 1.5	90.0 ± 3.0	86.9 ± 3.1	87.7 ± 7.6	84.9 ± 10.2
Resting Hemodynamics							
Systolic BP (mmHg)	99.7 ± 1.6 *	106.7 ± 2.0 *	103.5 ± 1.7	102.6 ± 1.7	101.4 ± 4.2	100.9 ± 9.4 *	107.3 ± 8.8 *
Diastolic BP (mmHg)	58.1 ± 1.7	59.4 ± 1.9	57.6 ± 1.3	57.9 ± 2.2	62.8 ± 5.0	57.3 ± 9.8	61.2 ± 6.5
HR (bpm)	78.9 ± 2.1	79.4 ± 2.6	77.4 ± 2.2	79.2 ± 3.4	83.9 ± 4.2	81.6 ± 11.3 *	73.1 ± 12.9 *
Fitness Measures							
Grip Strength (kg)	39.1 ± 3.2	45.7 ± 4.2	42.6 ± 3.6	44.9 ± 5.9	38.6 ± 4.6	35.0 ± 14.8 *	57.8 ± 18.9 *
Est.VO_2_max (mL/kg/min)	43.6 ± 0.7 *	54.4 ± 0.6 *	48.9 ± 1.0	49.5 ± 2.1	46.7 ± 3.1	49.2 ± 6.9 *	47.5 ± 5.8 *
DXA Measures							
FM (kg)	14.9 ± 1.8	12.6 ± 1.3	10.3 ± 0.6 *	15.2 ± 1.6 *	23.9 ± 3.8 *	13.5 ± 9.4	14.2 ± 5.5
FMI (kg/m^2^)	6.7 ± 0.7	5.6 ± 0.5	4.6 ± 0.2 *	6.9 ± 0.4 *	10.7 ± 1.3 *	6.6 ± 3.6	5.2 ± 2.1
BF (%)	31.6 ± 1.4 *	27.0 ± 1.7 *	25.1 ± 1.0 *	33.0 ± 1.7 *	40.1 ± 2.1 *	31.6 ± 8.1 *	24.6 ± 7.8 *
VAT (cm^2^)	39.6 ± 6.6	46.4 ± 3.4	30.9 ± 1.7 *	47.6 ± 4.7 *	79.3 ± 13.1 *	43.9 ± 32.2	41.2 ± 17.2
FFM (kg)	28.8 ± 1.8	32.6 ± 2.5	30.1 ± 1.8	30.2 ± 3.6	32.9 ± 4.2	25.5 ± 8.5 *	41.3 ± 9.7 *
FFMI (kg/m^2^)	12.9 ± 0.4	13.8 ± 0.4	12.9 ± 0.3 *	13.4 ± 0.6	14.8 ± 0.9 *	12.6 ± 2.1 *	14.8 ± 2.0 *
BIA Measures							
BMR (kcals/day)	1101 ± 38	1207 ± 62	1150 ± 45	1159 ± 92	1168 ± 92	1024 ± 30 *	1424 ± 55 *
Phase Angle (°)	5.2 ± 0.1 *	5.6 ± 0.1 *	5.4 ± 0.1	5.4 ± 0.2	5.4 ± 0.2	5.2 ± 0.1 *	5.9 ± 0.1 *
FM (kg)	12.3 ± 2.1	9.3 ± 1.3	7.0 ± 0.6 *	11.6 ± 1.5 *	22.8 ± 4.5 *	11.0 ± 1.7	10.4 ± 1.3
FMI (kg/m^2^)	5.5 ± 0.8	4.1 ± 0.5	3.1 ± 0.3 *	5.2 ± 0.5 *	10.1 ± 1.6 *	5.3 ± 0.7	3.9 ± 0.5
BF (%)	24.0 ± 1.9	18.8 ± 1.9	16.4 ± 1.1 *	24.4 ± 2.1 *	35.9 ± 3.1 *	23.4 ± 1.8	17.4 ± 2.0
VAT (cm^2^)	53.0 ± 10.0	37.8 ± 6.8	26.7 ± 2.7*	47.4 ± 8.1 *	106.4 ± 21.9 *	47.7 ± 8.5	40.7 ± 6.2
FFM (kg)	33.9 ± 1.8	38.8 ± 2.9	36.1 ± 2.1	36.5 ± 4.3	37.0 ± 4.3	30.3 ± 1.4 *	48.8 ± 2.6 *
FFMI (kg/m^2^)	15.3 ± 0.3	16.4 ± 0.5	15.5 ± 0.3	16.3 ± 0.7	16.8 ± 0.8	15.1 ± 0.3 *	17.5 ± 0.5 *

BMI, body mass index; RMR, resting metabolic rate; TC, total cholesterol; HDL, high-density lipoproteins; TRG, triglycerides; LDL, low-density lipoproteins; BP, blood pressure; Est.VO_2_max, estimated maximal oxygen consumption; DXA, dual-energy *x*-ray absorptiometry; VAT, visceral adipose tissue; FMI, fat mass index; FFMI, fat-free mass index; BIA, bioimpedance analysis; BMR, basal metabolic rate; HW, healthy weight (BMI < 85th percentile); OW, overweight (BMI ≥ 85th but <95th percentile); OB, obese (BMI ≥ 95th percentile); Child, 7–12 years; Teen, 13–17 years. One-way ANOVA was used to assess differences between sex, age group, and weight status. * Denotes significant difference within groups (i.e., girls vs. boys) in bold (*p* < 0.05).

**Table 2:** Comparison of body composition and RMR between DXA and BIA (means ± SEE).

		Body Fat (%)		FMI (kg/m^2^)		FFMI (kg/m^2^)		VAT (cm^2^)		RMR (kcal/day)	
	N	DXA	BIA	DXA	BIA	DXA	BIA	DXA	BIA	MG	BIA
All Youth	58	29.3 ± 1.1	21.4 ± 1.4	6.1 ± 0.4	4.8 ± 0.5	13.4 ± 0.3	15.9 ± 0.3	43.0 ± 3.7	45.4 ± 6.1	1104 ± 39 *	1244 ± 41 *
Sex											
Girls	30	31.6 ± 1.4	24.0 ± 1.9	6.7 ± 0.7	5.5 ± 0.8	12.9 ± 0.4	15.3 ± 0.3	39.6 ± 6.7	53.0 ± 10.0	1070 ± 48 *	1227 ± 44 *
Boys	28	27.0 ± 1.7	18.8 ± 1.9	5.6 ± 0.5	4.1 ± 0.5	13.8 ± 0.4	16.4 ± 0.5	46.4 ± 2.9 *	37.8 ± 6.8 *	1148 ± 64 *	1268 ± 75 *
Weight Status											
HW	37	25.1 ± 1.0 *	16.4 ± 1.1 *	4.6 ± 0.2 *	3.1 ± 0.3 *	12.9 ± 0.3 *	15.5 ± 0.3 *	30.9 ± 1.7	26.7 ± 2.7	1049 ± 44 *	1231 ± 48 *
OW	10	33.0 ± 1.7	24.4 ± 2.1	6.9 ± 0.4	5.2 ± 0.5	13.4 ± 0.6	16.3 ± 0.7	47.6 ± 4.7	47.4 ± 8.1	1153 ± 105	1248 ± 125
OB	11	40.1 ± 2.1	35.9 ± 3.1	10.7 ± 1.3	10.1 ± 1.6	14.8 ± 0.9	16.8 ± 0.8	79.3 ± 13.1	106.4 ± 21.9	1231 ± 94	1285 ± 101
Age Group											
Child	39	31.6 ± 1.3	23.4 ± 1.8	6.6 ± 0.6	5.3 ± 0.7	12.6 ± 0.3	15.1 ± 0.3	43.9 ± 5.2	47.7 ± 8.5	1038 ± 42 *	1113 ± 34 *
Teens	19	24.6 ± 1.8	17.4 ± 2.0	5.2 ± 0.5 *	3.9 ± 0.5 *	14.8 ± 0.5	17.5 ± 0.5	41.2 ± 4.0	40.7 ± 6.2	1234 ± 72 *	1541 ± 62 *

N, sample size; DXA, dual-energy *x*-ray absorptiometry; BIA, bioimpedance analysis; FMI, fat mass index; FFMI, fat-free mass index; VAT, visceral adipose tissue; HW, healthy weight (BMI < 85th percentile); OW, overweight (BMI ≥ 85th but <95th percentile); OB, obese (BMI ≥ 95th percentile); Child, age 7–12 years; Teen, age 13–17 years. One-way ANOVA was used to assess differences between sex, age group, and weight status. * Denotes significant difference between methods (i.e., DXA vs. BIA) overall (all youth) or within group (i.e., girls, HW, or Child) in bold (*p* < 0.05).

**Table 3:** Comparison of fasting vs. non-fasting BIA measures up to 2 h post-prandial (means ± SEE).

Variable	Timepoints											
	N	Baseline	N	0 min	N	30 min	N	60 min	N	90 min	N	120 min
Weight (kg)	58	47.1 ± 2.4	57	46.6 ± 2.3	57	46.7 ± 2.3	57	46.7 ± 2.3	57	46.6 ± 2.3	52	45.9 ± 2.5
BMR (kcals/day)	58	1155 ± 37	57	1143 ± 35	57	1144 ± 35	57	1144 ± 35	57	1147 ± 35	52	1144 ± 38
Phase Angle (°)	58	5.42 ± 0.08	57	5.43 ± 0.08	57	5.45 ± 0.08	57	5.44 ± 0.08	57	5.41 ± 0.08	52	5.40 ± 0.08
Fat Mass Analysis												
FM (kg)	58	10.8 ± 1.2	57	11.0 ± 1.3	57	10.9 ± 1.2	57	10.6 ± 1.3	57	12.0 ± 1.8	52	10.0 ± 1.2
BF (%)	58	21.4 ± 1.4 *	57	22.0 ± 1.4 *	57	21.9 ± 1.4 *	57	21.7 ± 1.4	57	21.4 ± 1.4	52	20.6 ± 1.3
VAT (cm^2^)	58	45.4 ± 6.1 *	57	46.2 ± 6.2 *	57	45.5 ± 6.6	57	45.2 ± 6.7	57	45.0 ± 5.9	52	40.7 ± 5.6 *
FMI (kg/m^2^)	58	4.7 ± 9.5	57	4.9 ± 1.5	57	4.8 ± 7.5	57	4.8 ± 4.5	57	5.4 ± 1.8	54	4.2 ± 7.5
Fat-Free Mass Analysis												
FFM (kg)	58	36.3 ± 1.7	57	35.8 ± 1.6	57	35.8 ± 1.6	57	35.8 ± 1.6	57	36.0 ± 1.6	52	35.8 ± 1.7
FFMI (kg/m^2^)	58	15.8 ± 6.3 *	57	15.7 ± 5.3	57	15.7 ± 7.3	57	15.7 ± 8.3	57	15.8 ± 3.3 *	54	15.2 ± 0.5 *
Body Water Analysis												
TBW (L)	58	26.6 ± 1.2 *	57	26.2 ± 1.2	57	26.2 ± 1.2	57	26.2 ± 1.2	57	26.3 ± 1.2 *	52	26.2 ± 1.3 *
ECW/TBW	58	0.3 ± 8.0 *	57	0.3 ± 8.0 *	57	0.3 ± 8.0	57	0.3 ± 8.0	57	0.3 ± 8.0 *	52	0.3 ± 8.0 *
TBW/LBM	58	73.2 ± 4.0	57	73.2 ± 1.0	57	73.2 ± 0.0	57	73.2 ± 1.0	57	73.2 ± 2.0	52	73.2 ± 4.0
%TBW	58	57.5 ± 1.0	57	57.5 ± 1.2	57	57.2 ± 1.0	57	57.3 ± 1.0	57	57.6 ± 1.0	52	58.1 ± 1.0

BIA, bioimpedance analysis; BMR, basal metabolic rate; FM, fat mass; BF, body fat; VAT, visceral adipose tissue; FMI, fat mass index; FFM, fat-free mass; FFMI, fat-free mass index; TBW, total body water; ECW/TBW, ratio of extracellular water to total body water; TBW/LBM, ratio of total body water to lean body mass; %TBW, percent of body weight accounted for by the weight of total body water. Repeated-measures ANOVA with Bonferroni adjustment was used to assess differences between timepoints. * Denotes the timepoint is significantly different from baseline in bold (*p* < 0.05).

## Data Availability

The data presented in this study are available on request from the corresponding author. The data are not publicly available yet due to continued data collection and identifiers have not yet been removed.
